# Aramid-wrapped CNT hybrid sol–gel sorbent for polycyclic aromatic hydrocarbons†

**DOI:** 10.1039/d2ra02659g

**Published:** 2022-06-20

**Authors:** Abdullah Alhendal, Randa Abd Almoaeen, Mohamed Rashad, Ali Husain, Fouzi Mouffouk, Zahoor Ahmad

**Affiliations:** Department of Chemistry, Kuwait University P. O. Box 5969, Safat 13060 Kuwait abdullah.alhendal@ku.edu.kw

## Abstract

This work describes the preparation of an analytical microextraction sorbent using a simple and versatile sol–gel hybrid composite, *i.e.*, aramid oligomers wrapping multi-walled carbon nanotubes (CNTs) covalently bonded to a porous silica network. To overcome the inherent shortcomings of the CNTs' solubility and dispersion in both organic phases and in the sol–gel solution, the outer surface of the CNTs was initially functionalized with carboxylic acid groups and then reacted with both aramid oligomers and 3-aminopropyl triethoxysilane (APTES). The obtained sorbent was characterized by FT-IR, scanning electron microscopy (SEM), and thermogravimetric analysis (TGA). Using sol–gel chemistry, the functionalized CNTs were coated onto SPME fibers and used in conjunction with GC-MS for the analysis of polycyclic aromatic hydrocarbons (PAHs) in water and soil samples. Excellent repeatability (run-to-run RSD% ∼ 8) and reproducibility (fiber-to-fiber RSD% ∼ 6) were achieved in addition to low LODs (0.10–0.30 ng mL^−1^) and noticeable recovery%. The present method of sorbent preparation led to enhanced thermal and chemical stabilities, a long sorbent lifetime and good affinity towards PAHs. Moreover, the present sorbent enhanced the extraction capability by more than 30% compared to that of commercially available PDMS counterparts.

## Abstract 2

This work describes the preparation of an analytical microextraction sorbent using a simple and versatile sol–gel hybrid composite, *i.e.*, aramid oligomers wrapping multi-walled carbon nanotubes (CNTs) covalently bonded to a porous silica network.

## Graphical abstract

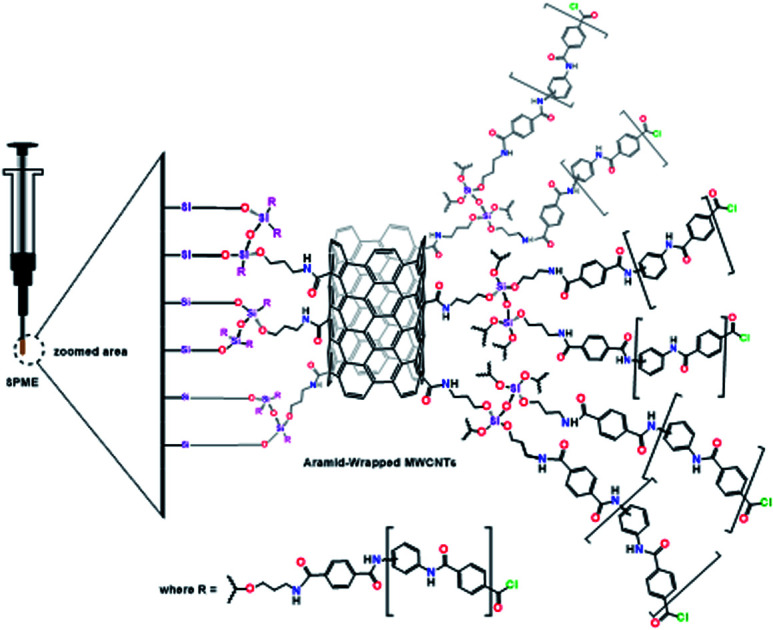

## Introduction

Polycyclic aromatic hydrocarbons (PAHs) are considered to be toxic and carcinogenic in nature; however, due to increased human activity, the level of light PAH pollution is dramatically increasing with time.^1^ Severe restrictions have now been placed on the content of PAHs in food, industrial products, and environmental samples.^2^ PAHs, especially the lighter kinds, are water-soluble, can bind to ash particulates and travel to surrounding areas even at trace-level concentrations, and can accumulate in soil and water systems.^3^ Well-known health hazards are associated with their presence in the environment, particularly near water resources and around oil refineries. PAHs are now considered priority pollutants by the United States Environmental Protection Agency (US-EPA).^4–6^ Accordingly, different extraction techniques, such as solid-phase extraction,^7–9^ SPME,^10,11^ dispersive micro-solid phase extraction,^12–14^ liquid–liquid extraction,^15^ and dispersive liquid–liquid micro-extraction combined with micro-solid-phase extraction, have been applied to isolate PAHs from water samples.^16^ Owing to the presence of PAHs in trace quantity in the matrix, an effective enrichment method should provide good accuracy, analytical microextraction performance, stability, selectivity, and loading capacity.^17–22^ However, some of these methods are time-consuming, lack automation or portability, and some consume large amounts of solvents. Therefore, innovations in solid-phase microextraction, which is a simple, portable, solvent-less and non-exhaustive method, are continuously needed to find new sorbents that are capable of providing enhanced analytical performance.

Single or multi-walled carbon nanotubes (CNTs) have been utilized as extractive sorbents due to their chemical stability, high surface area, and high surface-to-volume ratio.^23,24^ However, CNTs suffer from self-aggregation and stacking phenomena *via* π–π and van der Waals interactions, resulting in limited solubility in organic solvents. Therefore, various efforts have been made to functionalize the outer surface of CNTs to enhance and improve their solubility and dispersion in different solvents. For example, Sarafraz-Yazdi's group investigated the functionalization of CNTs with polyethylene glycol (PEG) polymeric tails to enhance their dispersion and compatibility in sol–gel matrices.^25^ Additionally, many modified CNTs with surfactants,^26^ amino acids,^27^ cyclodextrins,^28,29^ polythiophene,^30^ and polyaniline–polypyrrole^31^ have been prepared and their utility as microextraction sorbents has been evaluated. Moreover, nitrogen-doped CNTs with excellent analytical performance for the extraction of polychlorinated biphenyls (PCBs) have been described.^32^ In addition, other approaches have been utilized such as the electrodeposition of the layer-by-layer CNTs-ZIF-polyaniline sorbent for the extraction of PAHs, the use of oxidized CNTs as solid-phase extraction (SPE) sorbents for the extraction of polar analytes,^33^ and pristine CNTs for dispersive SPE,^34,35^ magnetic DSPE and molecularly imprinted polymers.^36^ The surface modification and functionalization of CNTs have been effectively used for the covalent linkage of CNTs with the microextraction media.^25,37,38^ Ahmad *et al.* proposed a convenient and robust method for the modification of acid-functionalized CNTs with aramid (Ar) oligomers *via* the aramid linkage (–ph–CO–NH–ph–).^39^ Ar-Chains on the CNT outer surface lead to better compatibility, miscibility, and dispersion of Ar-CNTs in organic solutions.

Sol–gel chemistry^40,41^ has offered a reasonable solution for the inherited drawbacks of conventional coating methods for SPME such as poor chemical and thermal stabilities. The sol–gel coating method has been widely investigated^42–48^ since it was introduced by Malik *via* hydrolytic and non-hydrolytic routes.^49,50^ Herein, an efficient, simple, and robust route has been developed for the preparation of a homogeneous organic–inorganic sorbent comprising aramid-wrapped carbon nanotubes covalently bonded into a sol–gel network, and a sol–gel binder (3-aminopropyl triethoxysilane, APTES) was used to play a dual role: (a) binding with the functional groups on the surface of the CNTs to create sol–gel “active” CNTs and (b) linking the aramid oligomers and the functionalized CNTs. This sol solution was then used for the preparation of the SPME sorbent.

## Experimental section

### Chemicals and materials

1,4- and 1,3-phenylene diamines and terephthaloyl chloride (TPC) were both of analytical grade (99% pure). Oxidized CNTs (outer diameter 8 nm, inner diameter 2–5 nm, length range 0.5–2 μm) with a purity of 95% were purchased from Nano Armor USA (4% oxidation). Pyrene, naphthalene, fluoranthene, acenaphthylene, phenanthrene, hexane, acetone, sodium hydroxide, 3-aminopropyl triethoxysilane (APTES), tetrahydrofuran, and anhydrous dimethylacetamide (DMAC) were purchased from Sigma Aldrich. Hydrochloric acid was obtained from J. T. Baker USA. Silica gel beads were obtained from Merck, while fused silica untreated fiber was purchased from Supelco.

### Apparatus

The morphology of the synthesized material was investigated using scanning electron microscopy (SEM). The samples were mounted on aluminum stubs, gold-coated using a Lecia ACE 200 Sputter coater, and then observed at 20 kV using a JCM-5700 SEM Carryscope (JEOL Company, Japan). Thermogravimetric analysis (TGA) was performed using a DTG-60 (DTA-TG instrument from Shimadzu, Kyoto, Japan). Measurements were taken at a heating rate of 10 °C min^−1^ from ambient temperature to 800 °C under a nitrogen atmosphere. FT-IR spectroscopy on the samples was performed on a Jasco spectrometer (Easton, MD, USA) within the frequency range of 400–4000 cm^−1^ using the pressed KBr disk technique. Chromatographic experiments were performed on a Shimadzu GC-2010 (Shimadzu, Kyoto, Japan) equipped with a mass spectrometry detector (GCMS-QP2010 Series). A Forte (SGE, Australia) GC capillary column (25 m × 0.25 mm ID and 0.25 μm thickness) was used for the separation. The total flow rate was maintained at 52.2 mL min^−1^. Helium was used as a carrier gas with a 1 mL min^−1^ flow rate. The injection port was set at 300 °C. The column temperature program: the initial temperature was 60 °C for one min, the hold-time temperature was then raised to 180 °C at a rate of 30 °C min^−1^, and afterwards at 30 °C min^−1^ till 280 °C and held for 5 min. Commercial SPME fiber (PDMS, coating thickness 100 μm, Supelco PA, USA) was generously provided by KU NUERS.

### Soil and seawater samples

Soil samples were collected from the gulf coast of Kuwait, crushed, and sieved with 100–200 mesh. The soil samples were used as a blank matrix and stored at 4 °C before use. Seawater samples were collected from Kuwait Gulf Bay using cleaned glass bottles and stored in a refrigerator. Crude seawater samples were filtered under vacuum through a 0.45 mm membrane filter and used later as a blank matrix. The blank soil and seawater samples were spiked with standard solutions of naphthalene, acenaphthylene, phenanthrene, fluoranthene and pyrene at a concentration of 0.3 mg L^−1^.

### Preparation of the sol–gel CNTs sorbent for SPME

#### Preparation of the aramid oligomers

**Scheme 1:**
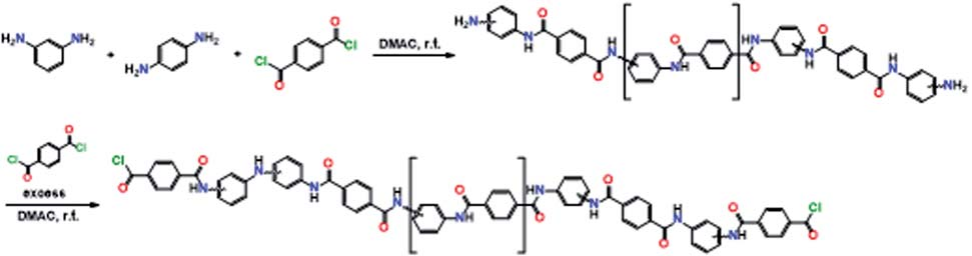
Synthesis of the aramid oligomers.

Aramid oligomers with COCl groups were prepared using a stoichiometric ratio of the monomers in the polymerization reaction of aramid, as described in the literature.^51^ A solution of TPC (0.985 mmol) in DMAC was taken in a 50 mL flask. A homogeneous solution (0.86 mmol) of 1,4- and 1,3-phenylene diamines, taken in a mole ratio of 35 : 65, in DMAC was then added dropwise to the TPC solution at 0 °C with stirring under complete anhydrous conditions (Scheme 1). The moles of amines used were 95% of the TPC in the reaction.

#### Preparation of the silanized carbon nanotubes (CNTs)

**Scheme 2:**
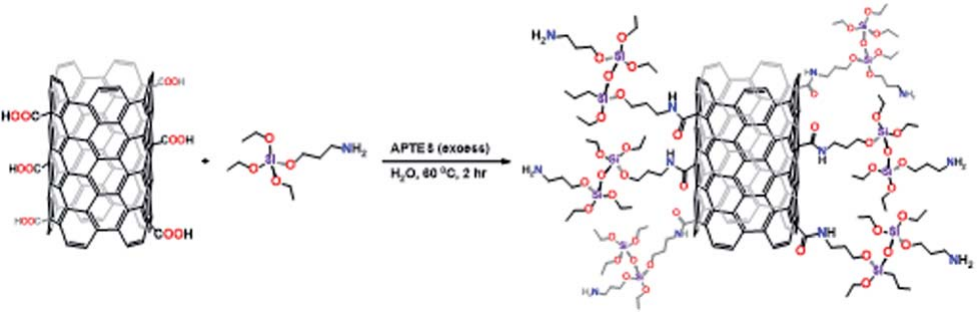
Synthesis of the silanized carbon nanotubes (CNTs).

10 mg oxidized CNTs were dispersed in 5.0 g DMAC and sonicated for 2 h to ensure homogeneity. Thereafter, 20 mg APTES was added, and the solution was vortexed and sonicated for 30 min. Finally, 20 mg water (containing 0.05% HCl) was added to the solution, followed by thorough vortexing. The solution was then heated at 60 °C with continuous stirring for 2 h, followed by the cooling of the resultant solution at room temperature (Scheme 2).

#### Preparation of the SPME sorbent

**Scheme 3:**
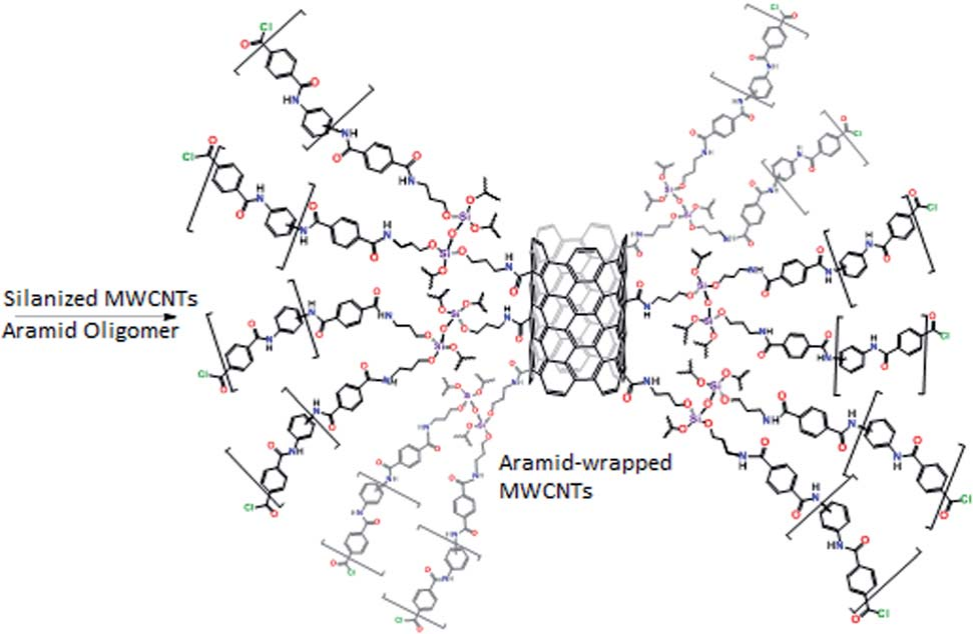
Synthesis of the aramid-wrapped CNTs.

**Fig. 1:**
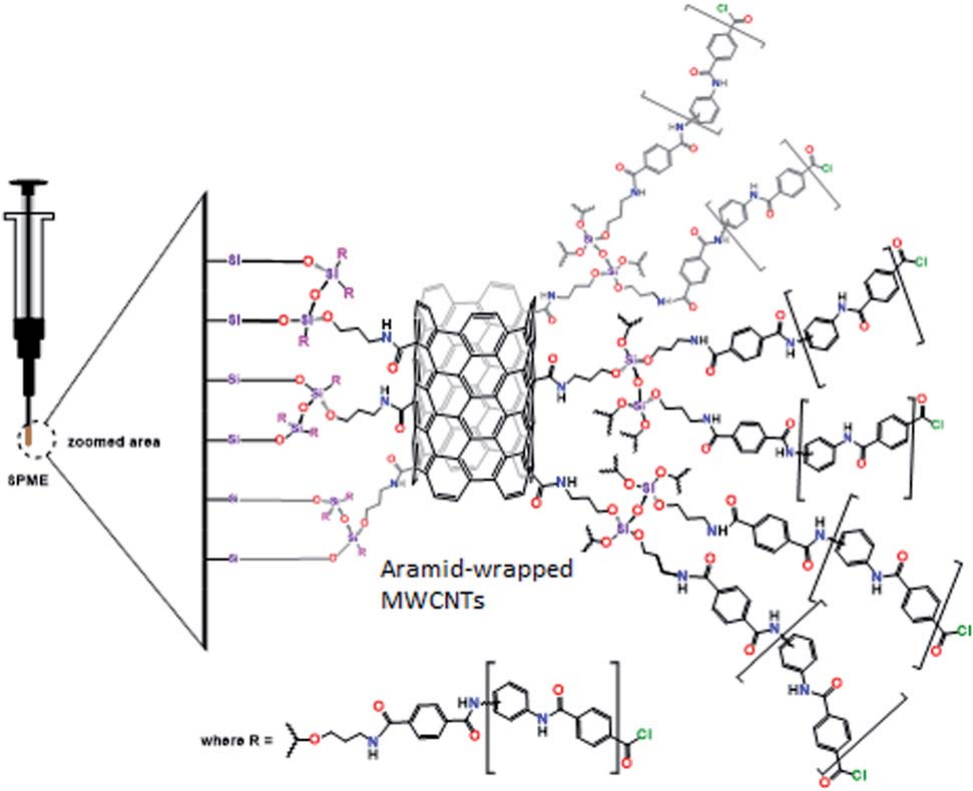
The aramid-wrapped CNT sorbent-coated SPME fiber.

An aliquot of 3 mL of the aramid oligomer solution was added to 5 mL of the silanized CNTs and the mixture was vortexed thoroughly (Scheme 3). The prepared sol-solution (Scheme S1†) was directly used to coat a 2 cm segment of the previously treated silica fiber that was vertically immersed in the freshly prepared sol-solution for 2 h and then placed in the oven at 60 °C for 24 h. The coated fiber was rinsed carefully with acetone and water, which was then thermally treated by placing it in a GC injection port at 200 °C for 1 h (Fig. 1).

### The procedure of the direct immersion SPME (DI-SPME)

A standard solution (100 μg mL^−1^) was used to prepare the PAH stock solution. PAH solutions (0.01 to 0.1 μg mL^−1^) were prepared by diluting the PAH stock solution with hexane. Prior to the extraction, the fiber was conditioned at 300 °C in the GC injection port for 1 h. The SPME coated fiber was immersed in 10 mL PAH sample solution for a predetermined extraction time. The coated fiber was then inserted in the GC inlet (at 300 °C for 6 min) for the thermal desorption of the extracted analytes.

### SPME for the soil and seawater samples

A spiked soil sample (2 g) was sonicated for 10 min and homogenized by vortexing with 10 mL hexane, which was added to extract the analyte from the soil sample. The mixture was then filtered through a filter membrane through three rounds of filtration. The resulting filtrate was then centrifuged for 10 min at 20k × *g*. The filtered and centrifuged supernatant was then evaporated under vacuum. The residue was then dissolved with 10 mL deionized water and vortexed and sonicated for 10 min each prior to extraction by immersion (DI-SPME).

## Results and discussion

### Characterization of the SPME sol–gel Ar-CNTs sorbent

**Fig. 2:**
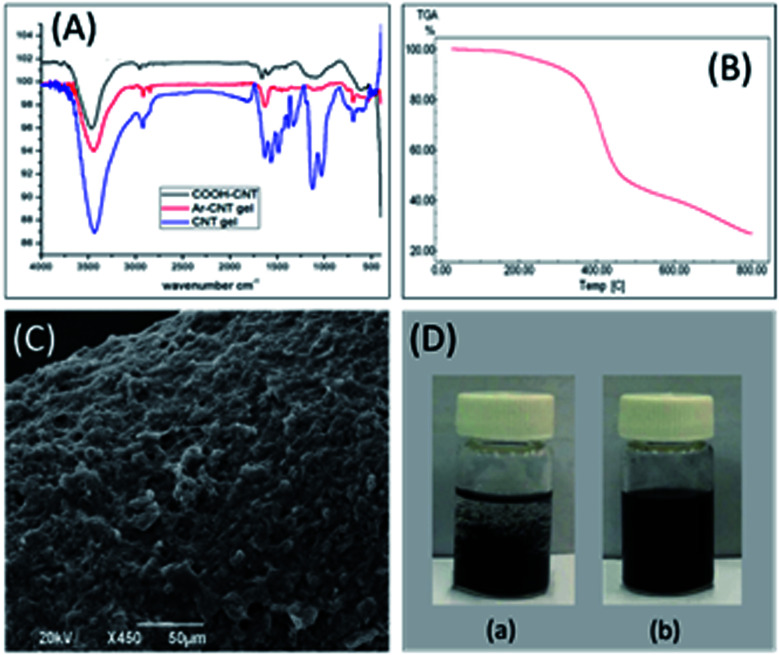
(A) FT-IR spectra of the acid-functionalized CNTs, APTES-functionalized CNTs, and Ar-wrapped sol–gel sorbent. (B) TGA thermogram of the Ar-wrapped sol–gel sorbent. (C) SEM image of the coated SPME fiber. (D) Homogeneity comparison between (a) the non-modified CNTs and (b) the Ar-wrapped CNTs in DMAC.

Initially, FT-IR analysis was carried out at all synthetic stages to confirm the formation of the composite material by monitoring the disappearance of the carboxylic acid functional group, *i.e.*, the C–O, O–H, and C=O stretching signals at 1160 cm^−1^, 3436 cm^−1^, and 1628 cm^−1^, respectively, in CNT-COOH^52,53^ (Fig. 2A, black color), and the presence of the amide group,^54^*i.e.*, N–H stretching at 3437 cm^−1^ and C=O stretching at 1488 cm^−1^, along with the existence of the APTES bands between 1500 cm^−1^ and 1250 cm^−1^ in CNT-APTES (Fig. 2A, red color). In addition, an increase in the absorption peak intensity at 1630 cm^−1^ attributed to the amide bond indicates the consumption of all –NH_2_ groups presented in the APTES sites in CNT-APTES upon the reaction with the acyl groups (COCl) of the aramid oligomer for the formation of the target sorbent, *i.e.*, aramid-wrapped CNTs (Fig. 2A, blue color).

Fig. 2B shows the TGA thermogram that was used to determine the thermal stability of the prepared sorbent. The temperature for maximum thermal decomposition was observed to be about 370 °C. A nearly 10% weight loss was noticed before 300 °C due to the evaporation of the remaining solvents and the by-products of the sol–gel process. The excellent thermal stability can be attributed to the presence of a stable aramid chain covalently linked with the CNTs^39^ and the linkage with the silica network produced during the sol–gel process. Additionally, various intermolecular interactions such as π–π and hydrophilic–hydrophobic interactions exist between the Ar oligomers and CNTs, which allow for the notable stability of the studied sorbent. Such results obtained from the TGA analysis support the use of this sorbent for GC applications.

Next, scanning electron microscopy images were taken to examine the morphology of the prepared coating. The micrograph shown in Fig. 2C indicates the rough and homogeneous morphology of the sol–gel sorbent. The sol–gel coating method endows an excellent porous morphology to the prepared sorbent. The reproducibility of the coating thickness was evaluated by preparing triplicates of the presented coating with excellent reproducibility of the coating thickness (fiber-to-fiber RSD% < 5.2), as measured using the SEM images. The homogeneity of the prepared aramid-wrapped CNTs in DMAC solution is clearly shown in Fig. 2D when compared to the acid-functionalized CNTs, which can be attributed to the combination of the aramid wrapping and APTES functionalization of the CNTs.

### Optimization of the SPME fiber and conditions

#### Extraction time

**Fig. 3:**
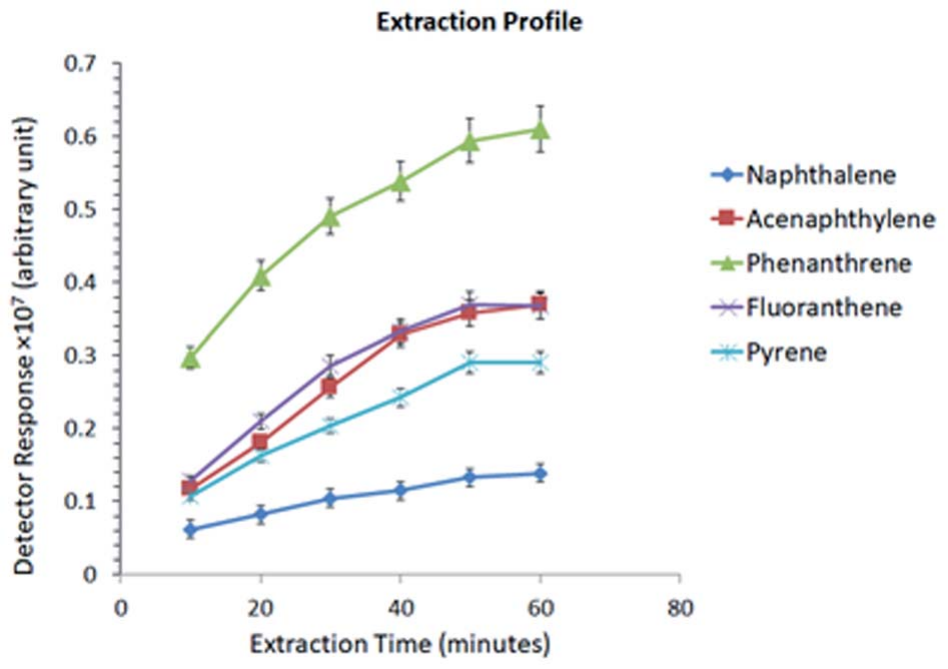
Extraction profile at different extraction intervals.

The optimum extraction time or the time of direct immersion of the SPME sorbent into the sample was studied at different time intervals, ranging from 10 to 60 min. Since SPME is an equilibrium-based technique, the extracted amount of the analyte can be related to various factors such as the sorption–desorption mechanism, coating thickness, and loading capacity, which have been examined for the present sorbent. Fig. 3 shows that the extraction equilibrium was reached within a reasonable time interval (50 min).

#### Desorption conditions

To optimize the desorption conditions, the desorption time and temperature were investigated. The desorption temperature was evaluated between 150 and 300 °C. Typically, in SPME-GC, thermal desorption breaks the intermolecular forces between the extracted analytes and the sorbent; however, complete desorption within the operational temperature must be accomplished and it was observed that the extracted analytes were completely desorbed at 280 °C. To ensure complete analyte desorption and to avoid any sample carryover, all the SPME desorption experiments were carried out at 300 °C. The desorption time was also evaluated among different desorption periods, and it was found that within 5 min, the PAHs were desorbed.

#### Coating thickness

**Fig. 4:**
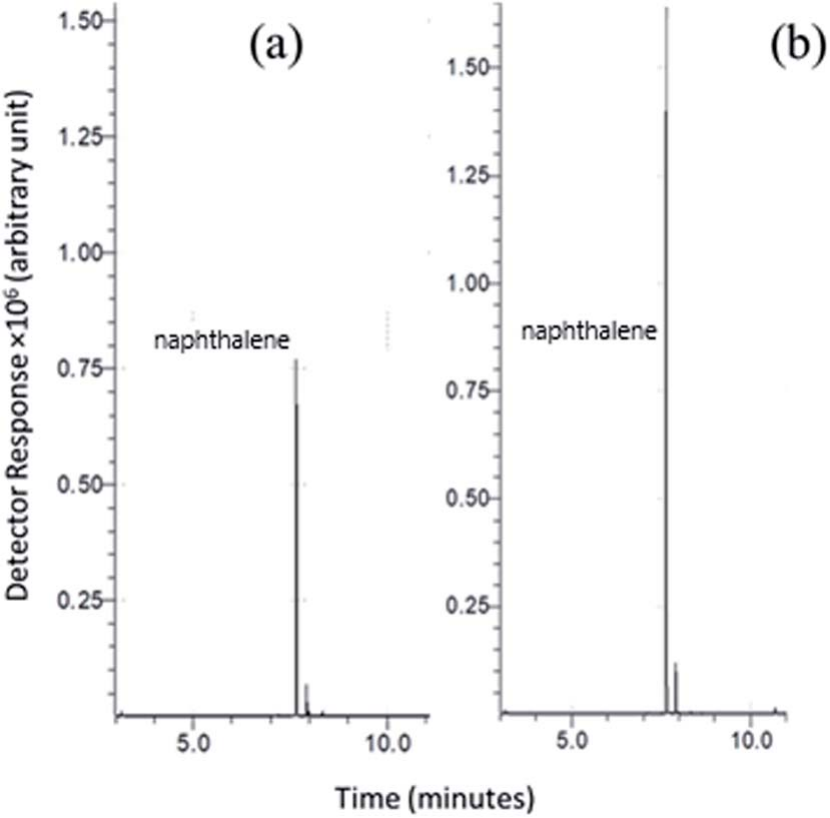
SPME-GC chromatogram of the Ar-wrapped sol–gel sorbent: (a) single layer and (b) triple layer.

The coating thickness is one of several critical parameters that must be evaluated carefully. The loading capacity of the SPME sorbent increased with the coating thickness, but it can also affect the desorption mechanism, leading to incomplete desorption or sample carryover. The coating thickness in this study was increased by replicating the coating of the layer-by-layer replication. The extraction of acenaphthylene was then studied to compare the extraction capability of a monolayer *versus* a three-layer coating. Fig. 4 shows a notable enhancement when the three-layer sorbent was used (117% enhancement in peak area). A fourth layer was tried and led to a more difficult desorption mechanism; thus, only a three-layer coating was used for all the experimental investigations.

#### Sorbent reusability

**Fig. 5:**
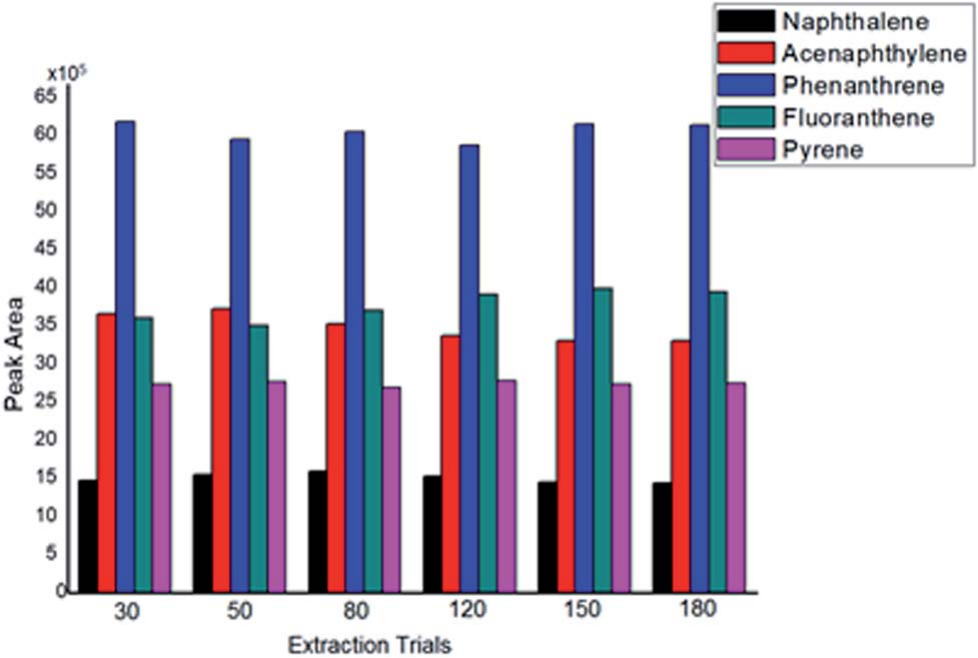
Comparison of the analytical performance of Ar-CNT in repeated trials (concentration of PAHs, 5 μg L^−1^; desorption temperature, 280 °C; desorption time, 5 min; extraction time, 50 min).

Reusability is a critical factor in determining the robustness of a sorbent. To determine whether the synthesized sorbent could be efficiently reused, the sorbent was washed two times with toluene before subsequent use to ensure that no PAHs remained on the surface of the SPME sorbent. The results indicate that the Ar-wrapped sol–gel sorbent could be used without obvious loss in its extraction efficiency (Fig. 5). Strong covalent linking between the sol–gel components resulted in the good chemical and mechanical stability and reusability of the sorbent.

#### Method validation

**Fig. 6:**
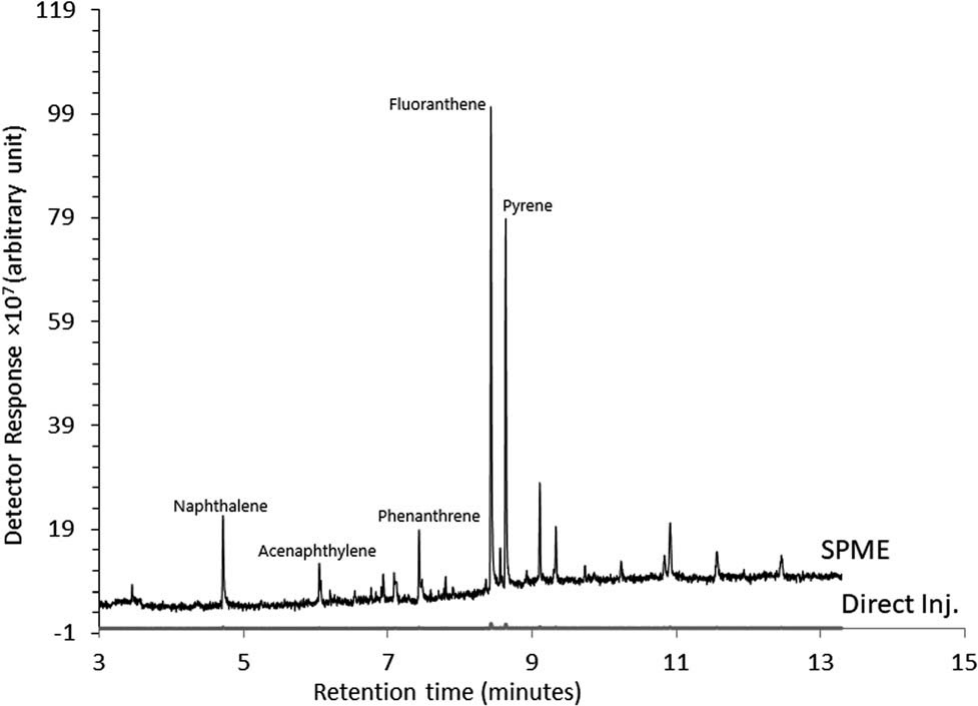
SPME-GC chromatogram of five PAHs extracted from the water sample using a conventional direct immersion method and enrichment by the Ar-CNT SPME compared to crude sample analysis (Direct Inj.) under the same experimental conditions (concentration of PAHs, 0.5 mg L^−1^; desorption temperature, 280 °C; desorption time, 5 min; extraction time, 50 min).

Fig. 6 demonstrates a comparison of SPME-GC for a PAH mixture at 0.5 mg L^−1^ compared to a directly injected sample prior to enrichment and at the same concentration level. The directly injected sample showed no significant response, while the Ar-CNT SPME allowed for enhanced detectability and analysis (Fig. S1†).

**Fig. 7:**
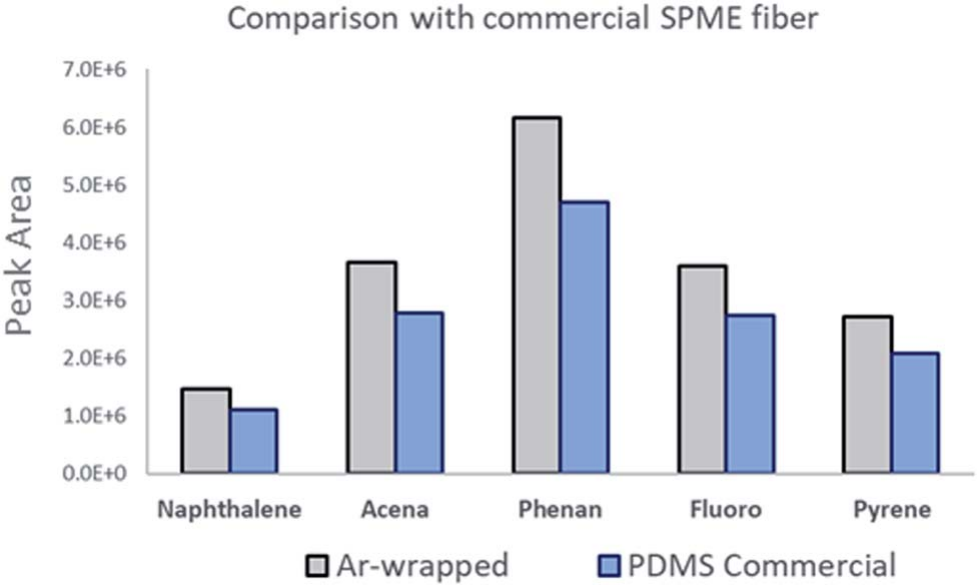
Illustration of the peak area comparison between the Ar-wrapped CNT sorbent and the commercial PDMS sorbent.

Fig. 7 illustrates a comparison between the prepared sorbent and a commercially available SPME sorbent (PDMS, 100 μm thickness). About a 30% enhanced peak area was observed and obtained using the Ar-wrapped CNT sorbent compared to the commercial PDMS sorbent.

Table 1 shows the analytical parameters obtained by the Ar-CNT-coated SPME fiber. The linearity, repeatability, reproducibility, limits of detection (LODs), and limits of quantitation (LOQs) are presented. Excellent run-to-run RSD% values (<8.5) revealed the repeatability of the present sorbent for the extraction of a mixture of PAHs carried out from triplicate measurements. Fiber-to-fiber reproducibility was evaluated using 3 fibers coated with the Ar-CNTs sorbent; the RSD% for the peak area obtained by triplicate measurements was found to be below 6. The LOD levels (0.1 to 0.3 ng mL^−1^) confirmed the good sensitivity of the Ar-CNT sorbent toward these carcinogenic pollutants due to the various intermolecular interactions. Excellent recovery% was achieved using the present sorbent in the range of 91 to 103. For a non-exhaustive technique such as SPME, the concept of recovery is related to the extraction process rather than the actual removal of the analyte from the sample matrix since non-exhaustive techniques extract at minimum micrograms or even nanograms of the analyte. For recovery% evaluation, the original sample concentration was compared to the sample concentration revealed by the extraction process, which is described by the following equation:*n* = *K*_fs_*V*_f_*C*_o_where *n* is the amount extracted, *K*_fs_ is the distribution coefficient, *V*_f_ is the fiber volume and *C*_o_ is the original sample concentration.

**Table 1:** Analytical parameters for the SPME-GC experiments with PAHs^a^

Analyte	LOD (S/N = 3)^b^	LOQ (S/N = 10)^b^	*R* ^2^	*R*%	RSD%^c^
Naphthalene	0.10	0.33	0.999	98	4.4
Acenaphthylene	0.30	0.99	0.998	103	2.3
Fluoranthene	0.30	0.99	0.998	87	6.0
Pyrene	0.30	0.99	0.998	91	8.1
Phenanthrene	0.20	0.66	0.999	92	4.4

a Working range: 0.01 to 0.1 μg mL^−1^.

b LODs and LOQs (ng mL^−1^).

c Run-to-run repeatability.

### Applicability to soil and seawater samples: a proof of concept

**Fig. 8:**
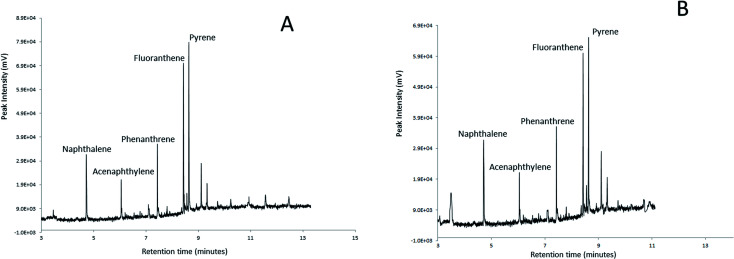
SPME-GC chromatograms for naphthalene, acenaphthylene, phenanthrene, fluoranthene and pyrene spiked in soil (A) and seawater (B) samples extracted using the Ar-CNTs sorbent.

To judge the applicability in real samples, a proof-of-concept experiment was conducted by the extraction of naphthalene, acenaphthylene, phenanthrene, fluoranthene and pyrene from soil and seawater samples. Fig. 8 illustrates the SPME-GC chromatograms of the PAH mixtures extracted from (A) the soil sample and (B) the seawater sample (in triplicate measurements).

## Conclusion

A hybrid, stable, and tunable sol–gel-based sorbent served as an SPME fiber coating. CNTs were efficiently wrapped with aramid oligomers to provide good miscibility and homogeneity in organic phases. Herein, a sol–gel binder was implemented to provide a dual role: covalent functionalization for the outer surface of the CNTs and sol–gel activation. The presence of various intermolecular interactions such as π–π stacking, hydrophilic, and hydrophobic interactions in the prepared sorbent allowed for noticeable affinity toward PAHs in water and soil samples. The prepared SPME sorbent allowed for acceptable levels of the obtained analytical parameters. Excellent sorbent reproducibility (fiber-to-fiber), SPME repeatability (run-to-run), recovery% and detection enhancement were achieved along with a significant lifetime. The method of preparation described in this work can be further utilized for the attachment of different organic moieties into sol–gel organic–inorganic hybrid composites for tunable sorbent design.

## Conflicts of interest

There are no conflicts to declare.

## Supplementary Material

RA-012-D2RA02659G-s001
