# Supplementary material for: Aramid-wrapped CNT hybrid sol–gel sorbent for polycyclic aromatic hydrocarbons
Source: RSC Adv. 2022 Jun 20;12(28):18077–83. doi: 10.1039/d2ra02659g (PMC9207600; doi:10.1039/d2ra02659g)
Supplement: RA-012-D2RA02659G-s001 [file RA-012-D2RA02659G-s001.pdf]

Electronic Supplementary Material (ESI) for RSC Advances.
This journal is © The Royal Society of Chemistry 2022

Supplementary Materials

Aramid-Wrapped CNTs Hybrid Sol-gel Sorbent for Polycyclic Aromatic Hydrocarbons

Abdullah Alhendal*, Randa Abd Almoaeen, Mohamed Rashad, Ali Husain, Fouzi Mouffouk, Zahoor Ahmad

Department of Chemistry, Kuwait University, P.O. Box 5969, Safat, 13060, Kuwait.

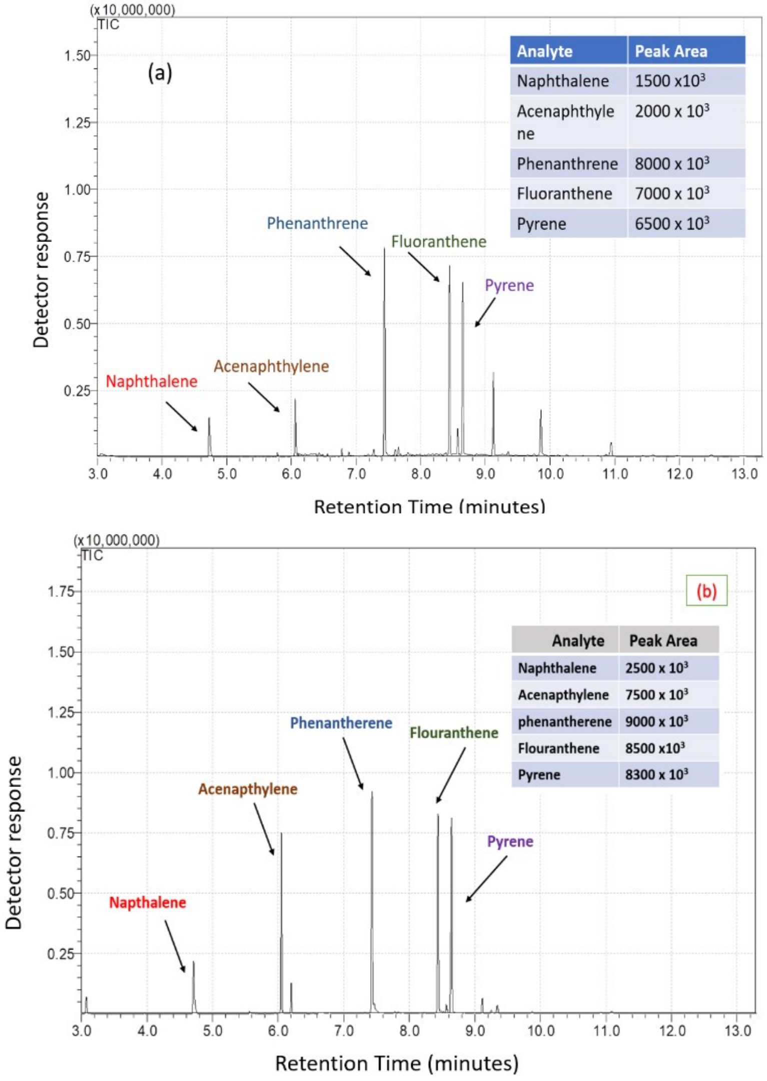

Fig. S1: SPME-GC-MS chromatograms for the extraction of 0.05 µg/L PAHs mixture by (a) sol-gel APTES (no Ar-MWCNTs) sorbent (b) sol-gel Ar-CNTs sorbent.

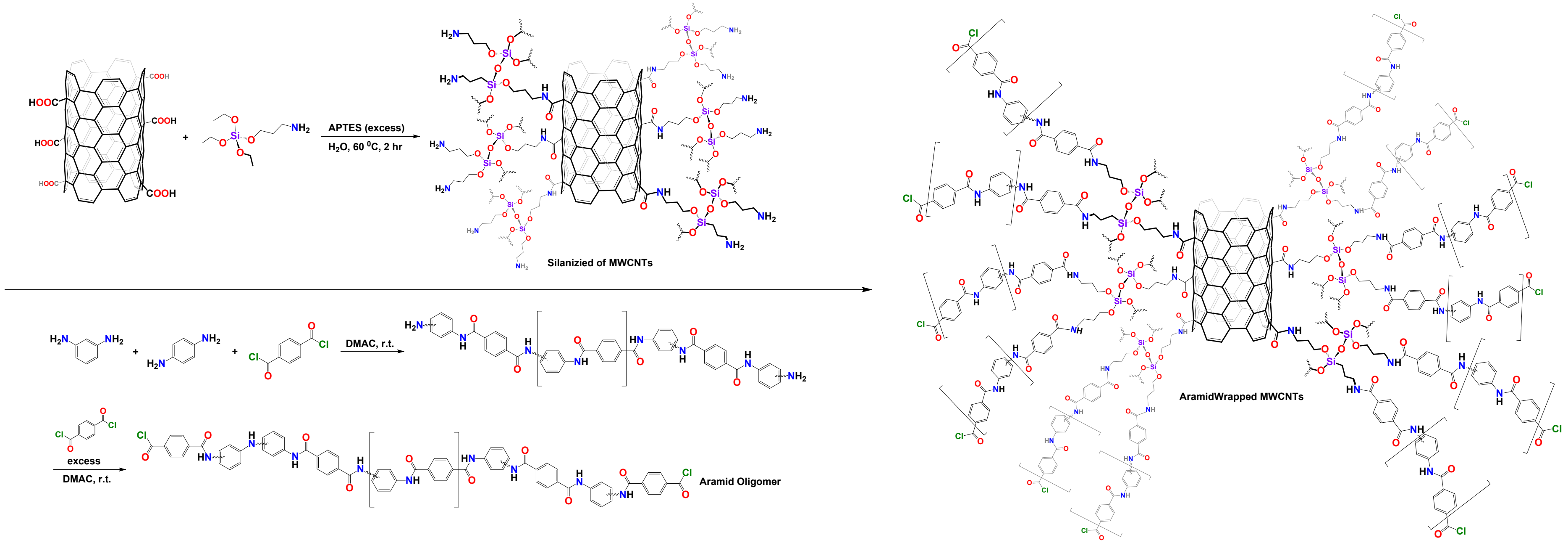

Scheme S1. Synthesis of Aramid-wrapped CNTs sorbent
